# Supplementary material for: A parcellation scheme of mouse isocortex based on reversals in connectivity gradients
Source: Netw Neurosci. 2023 Oct 1;7(3):999–1021. doi: 10.1162/netn_a_00312 (PMC10473268; doi:10.1162/netn_a_00312)
Supplement: Supplementary file 1 [file netn-7-3-999-s001.pdf]

Guyonnet-Hencke, T. & Reimann, M.W. (2023). Sleep spindles in primates: A Parcellation Scheme of Mouse Isocortex Based on Reversals in Connectivity Gradients. *Network Neuroscience*, Advance publication. https://doi.org/10.1162/netn_a_00312.

Supplementary Material

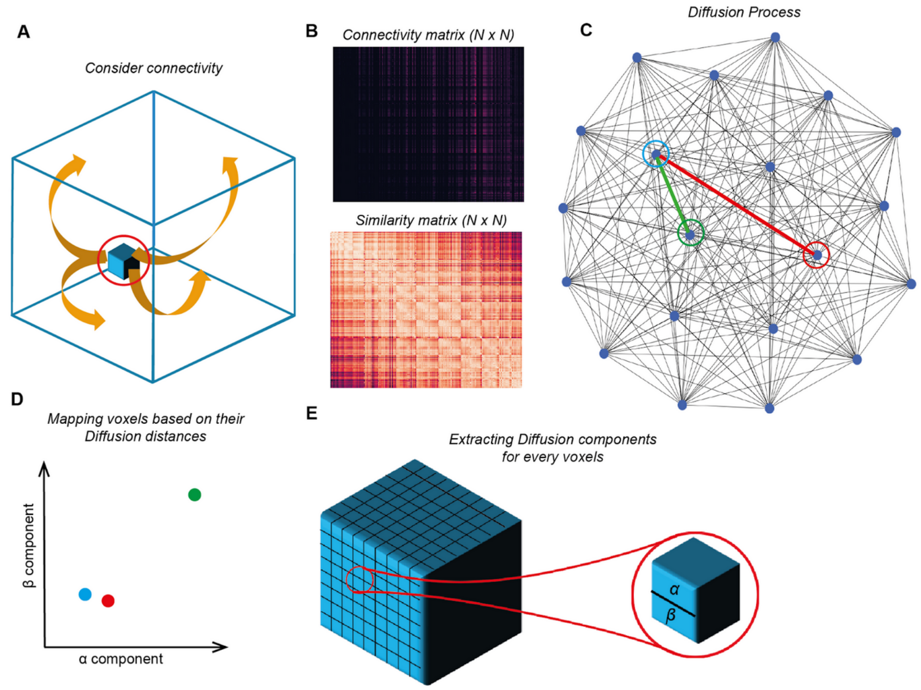

Figure S1: **Framework of the diffusion embedding.** From a connectivity matrix between source voxels and target voxels, we build a similarity matrix based on how the voxels' connectivity profile is similar (A-B). On this matrix, we run the diffusion process which will strengthen high connected pathways (red) and weaken connected pathways (green) (C). This process allows to reveal an embedded geometrical space of connectivity, where strongly connected voxels are close to each and in opposite poorly connected voxels are far from each other (D). To flatten this geometrical space, we therefore extract the 2 dimensions associated with the 2 strongest eigenvectors, which we call alpha and beta components (E).

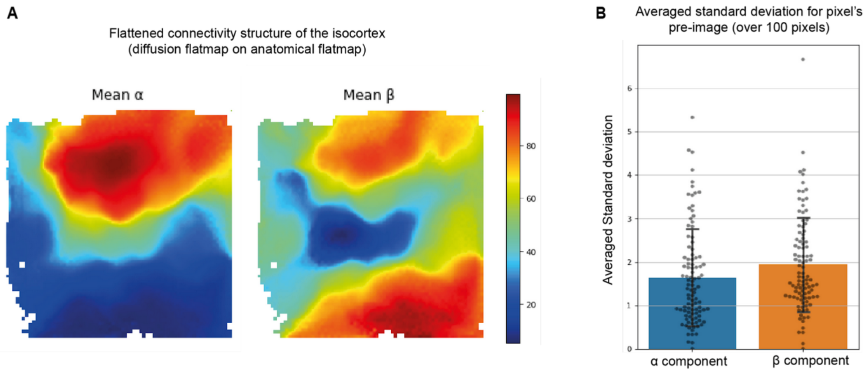

Figure S2: **Projecting values of connectivity components to two dimensions.** (A) Flattened reconstruction of the connectivity structure of the mouse isocortex by averaging the diffusion components of voxels corresponding to a pixel. (B) Standard deviation of the diffusion components of voxels corresponding to a pixel, averaged over 100 pixels.

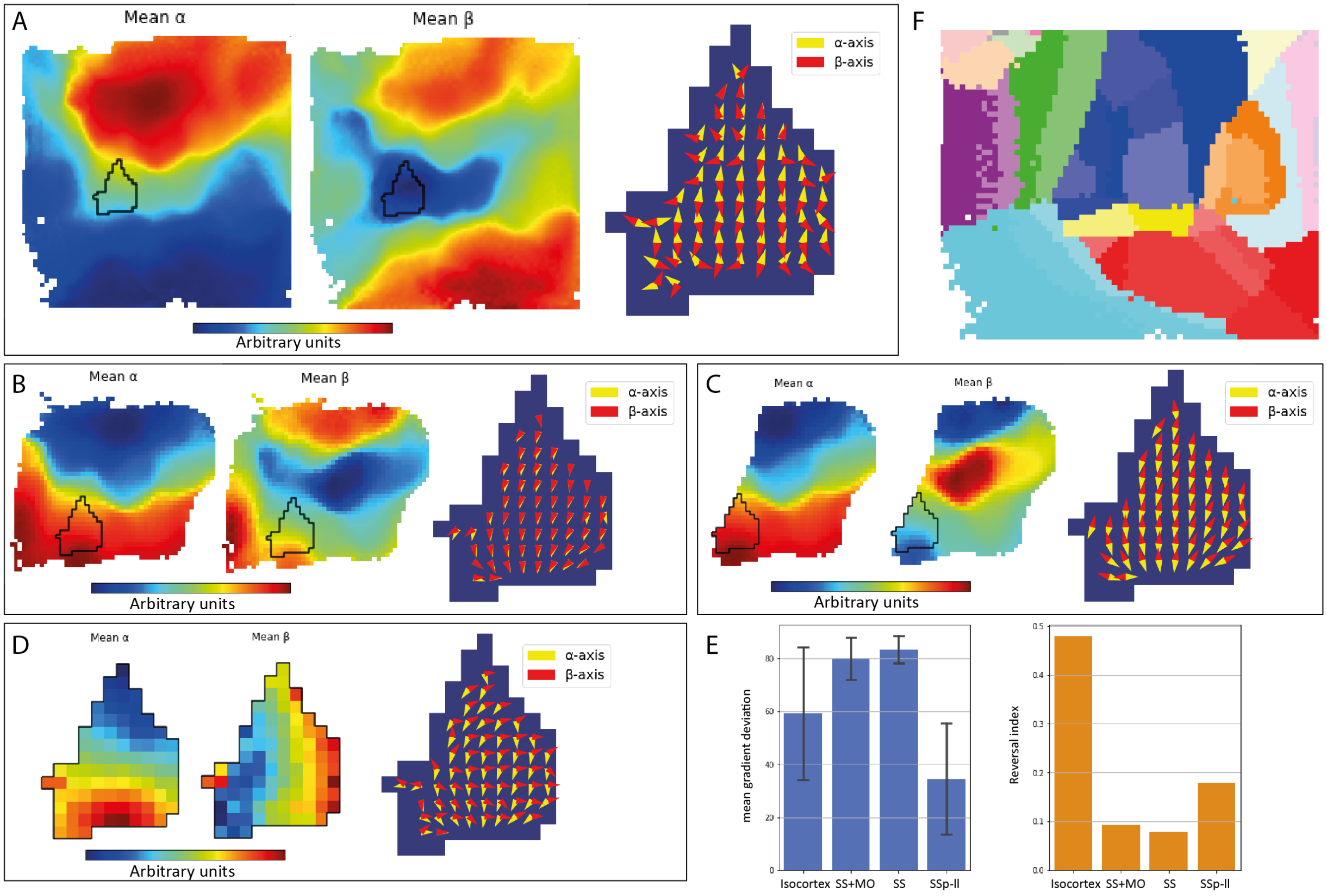

Figure S3: **Diffusion embedding with different spatial contexts.** A: Diffusion embedding performed on the whole isocortex, SSp-ll is annotated by the black line. B: Diffusion embedding of the Somatosensory and Somatomotor areas (blue and green in the parcellation in E.) C: Diffusion embedding of the Somatosensory areas (blue in the parcellation E.) D: Diffusion embedding of the SSp-ll. E: Mean gradient deviation (error bars = standard deviation) and reversal index of SSp-ll at different scales. Even though the connectivity structure of SSp-ll is homogeneous and continuous according to the gradient deviation and reversal index, it can only be revealed by isolating it after successive splits, hence the motivation to split the isocortex several time around gradients' reversals detected thanks to our method. F: Hierarchical organization of the AIBS CCFv3.

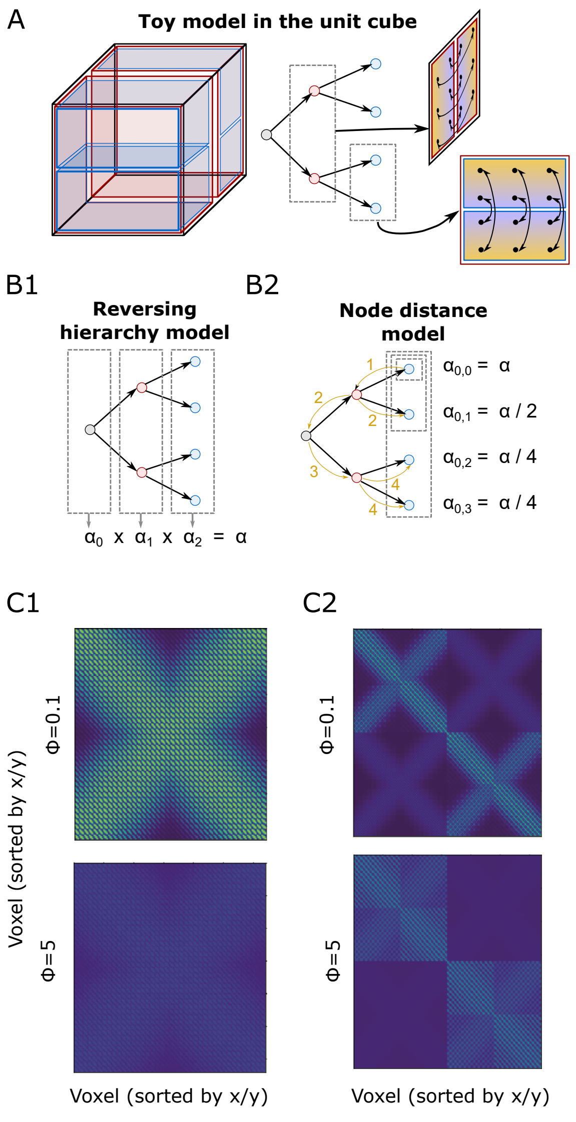

Figure S4: **Toy models for the evaluation of the algorithm.** A: Left: A model hierarchically splits the unit cube into equal quadrants. Right: The value of the strongest connectivity component is then prescribed as a linear gradient inverting at each border. Connectivity is then based on similarity of the prescribed connectivity components. B: The method yields one connection matrix at each hierarchy level. We construct a merged matrix by either multiplying the matrices at all levels (*reversing hierarchy model*, B1), or by considering the matrix at the lowest level and dividing the values in individual submatrices by the path distance between the nodes they represent in the hierarchy graph (*node distance model*, B2, distances from $\alpha_{0,0}$ indicated in yellow). C: Connection matrices of the two models with low ($\phi = 0.1$) and high ($\phi = 5$) noise added. D: More complex models are randomly generated by recursively splitting the space in two with lines drawn at random angles. At each split, the points on one side of the line are set to project towards locations at the mirrored opposite side (green arrows for an exemplary source indicated by a red dot). The range around the destination decreases, but strength increases with successive splits. White noise at $\phi = 0.5$ was added to projection strengths of each voxel pair. E: Connection matrix of the random parcellation in D. Sorted by x, y coordinates (E1) or by region (E2).

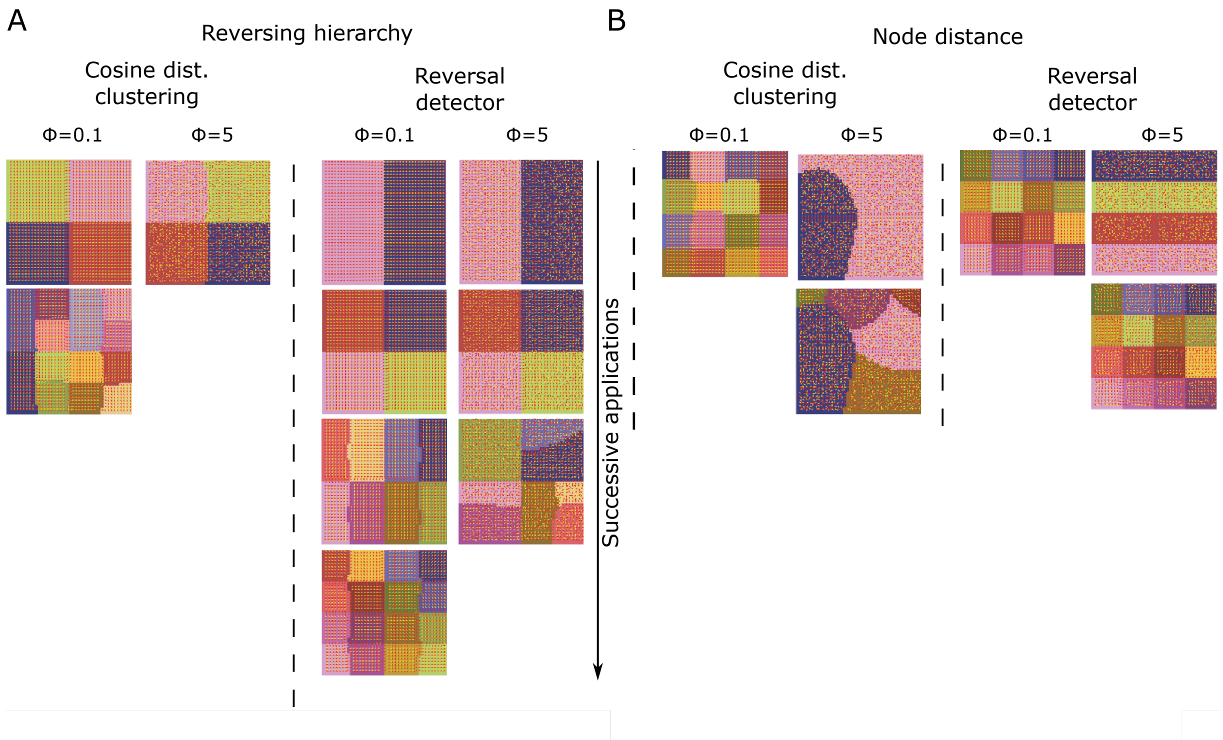

Figure S5: **Intermediate results reached while splitting toy models.** For the reversing hierarchy (A) and node distance (B) models as indicated in Fig. 3.

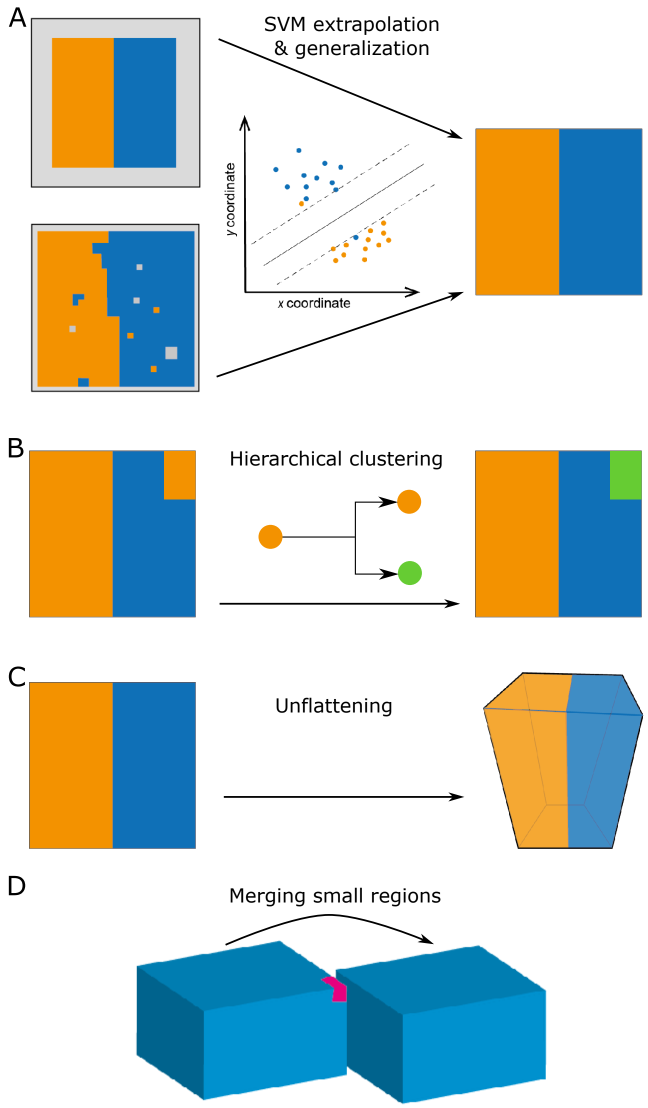

Figure S6: **Post-processing of the initial parcellation.** Post-processing steps, as indicated in Fig. 2A, that improve the initial parcellation. A: The initial parcellation of reversal detection (top) has many unlabelled pixels (grey) in the periphery. The parcellation of cosine distance clustering (bottom) is noisy and also has unlabelled pixels. A support vector machine is trained that predicts the region label from the location of a pixel. The prediction of the SVM features straighter region boundaries and labels for all pixels. B: Occasionally, the same region label is applied to spatially non-continuous patches. For a given label, hierarchical clustering is applied to the pairwise distances of the location of associated pixels. This splits up non-continuous regions. C: The parcellation is unflattened, i.e. projected back into 3d space by looking up all voxels that are associated with a given pixel. D: Regions below a volume threshold are merged with their nearest neighbor.

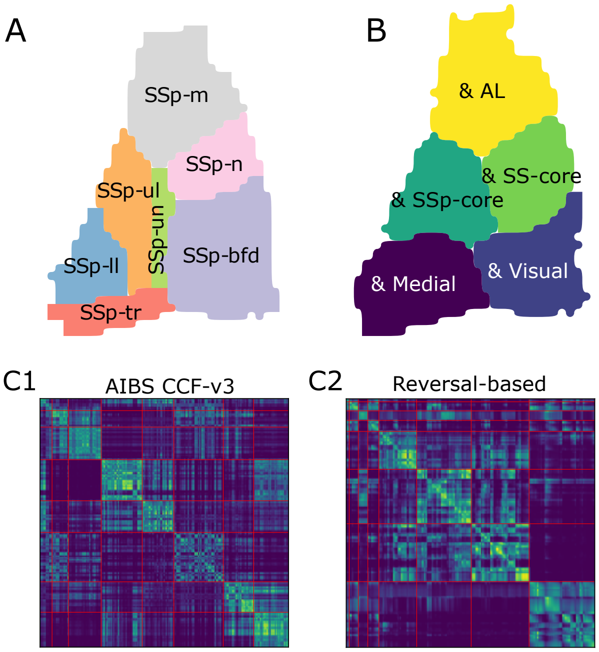

Figure S7: **Comparison of the parcellation of somatosensory regions.** A: Parcellation of primary somatosensory regions, as defined in the established AIBS CCF. B: Intersections of primary somatosensory regions in AIBS CCF with regions at the first hierarchy level of our reversal-based parcellation. C: Matrix of connections strengths between cortical locations. C1: Rows and columns sorted by high-level regions of the established parcellation (as shown in Fig. 4E). C2: Sorted by regions in the first hierarchy level of the reversal-based parcellation. Red lines indicate breaks between regions.
